# Is MS Intention Tremor Amplitude Related to Changed Peripheral Reflexes?

**DOI:** 10.5402/2011/192414

**Published:** 2011-09-26

**Authors:** Peter Feys, Werner Helsen, Stephan Ilsbroukx, Tom Meurrens

**Affiliations:** ^1^Departments of Biomedical Kinesiology and Rehabilitation Sciences, Katholieke Universiteit Leuven, 3000 Leuven, Belgium; ^2^REVAL, PHL University College Hasselt and BIOMED, University of Hasselt, 3590 Diepenbeek, Belgium; ^3^National MS Center Melsbroek, 1820 Melsbroek, Belgium

## Abstract

Intention tremor is related to lesions in the cerebellum or connected pathways. Intention tremor amplitude decreased after peripheral arm cooling in patients with multiple sclerosis (MS), likely caused by a reduction of muscle spindle afferent inflow, while amplitude increased when muscle spindles were artificially stimulated by tendon vibration. This study investigated the contribution of peripheral reflexes to the generation of MS intention tremor. Tendon reflexes of biceps, triceps, and brachioradialis, muscles were measured, using an electromechanical triggered reflex hammer. MS patients with (*n* = 17) and without (*n* = 17) upper limb intention and 18 healthy controls were tested. Latency of brachioradialis, biceps, and triceps tendon reflexes was greater in MS patients with tremor than in healthy controls and MS patients without tremor (except for the triceps reflex). Peak and peak-to-peak amplitude were not different between groups. It is concluded that tendon reflexes were delayed but not enlarged in MS patients with tremor.

## 1. Introduction

Tremor in multiple sclerosis (MS) is a low-frequency action tremor with the clinical picture often being a combination of postural and intention tremor [1]. Intention tremor, clinically defined as an increase in tremor amplitude during visually guided movements towards a target at the termination of the movement, is related to lesions in the cerebellum and/or connected pathways in the brain stem and is often synonymously used with cerebellar tremor [1, 2]. 

Cerebellar tremor is suspected to be related to unstable central motor pathways and a malfunction of feedforward loops within the central nervous system, especially the cerebellum [3–5]. A feedforward system predicts the consequences of a movement, even prior to movement onset. As such, fine-tuning of movements can occur prior and during movement execution, and time delays inherently associated with sensory feedback can be overcome. In contrast, the motor performance is more dependent on feedback information when a malfunction in the feedforward system is present. This may explain the susceptibility of cerebellar tremor to peripheral factors such as mechanical loading. Tremor amplitude and frequency were shown to be modulated by mechanical loads, which indicates the involvement of stretch-elicited peripheral feedback mechanisms in the manifestation of cerebellar tremor [6, 7]. In support of this view, load-compensating tasks, evoking sudden stretch, induced an increase of tremor in cerebellar patients [8]. The tremor increase was suggested to be caused by delayed and enlarged long-latency stretch reflexes which have been observed before in patients with cerebellar tremor [5, 8, 9]. Other studies manipulated the sensory input to the central nervous system. A reduction of cerebellar tremor during handwriting has been found after the application of an ischaemic block to the arm [10]. Previous research in MS patients showed that tremor was influenced by sensory information. Intention tremor amplitude decreased during visually-guided movements after peripheral cooling, likely caused by a reduction of the muscle spindle afferent inflow [11]. In contrast, overall tremor amplitude increased during memory guided movements when muscle spindles were artificially activated by means of tendon vibration [12]. Similarly, an increase of tremor and in coordination had been reported during high-frequency tendon vibration in patients with cerebellar dysfunction [13]. The effects of both cooling and tendon vibration on tremor amplitude are likely related to the changed activity of the muscle spindles influencing the reflex arc and may indirectly suggest a contribution of abnormal peripheral reflexes to the generation of tremor in MS.

The present study investigated the contribution of reflex activity to intention tremor amplitude in patients with MS, by measuring tendon reflexes (T-reflexes) which are considered as phasic stretch reflexes [14]. Specifically, latency, peak amplitude, and peak-to-peak amplitude of biceps brachii, triceps brachii, and brachioradialis tendon reflexes were studied in MS patients with intention tremor, MS patients without tremor, and healthy control subjects. These 3 groups were compared in order to differentiate general MS-related deficits such as decreased nerve conduction velocity from specific reflex abnormalities due to the lesions causing tremor. It was hypothesised that MS patients with intention tremor would show delayed and enlarged T-reflexes compared to MS patients without intention tremor and healthy control subjects.

## 2. Methods

### 2.1. Subjects

17 MS patients with intention tremor (9 men and 8 women; mean age 48.1 years with range 33–65; 15 right handed, 2 left handed) and 17 MS patients without intention tremor (6 men and 11 women; mean age 49.1 years with range 32–72; all right handed) were selected from patients with clinically definite MS by neurologists of the Belgian National MS Centre in Melsbroek. In both groups, arms showing clinically detectable spasticity, muscle paresis (score below 4+ on the Medical Research Council), and sensory loss were excluded. Overall disability was rated using the expanded disability status scale (EDSS) [15], obtained from the medical files of the patient. The clinical characteristics of both patient groups are summarised in Table 1. In addition, a healthy control group of 18 persons (6 men and 12 women; mean age 37.2 years with range 22–56; 15 right handed, 3 left handed) without known neurological deficits was selected. The study was conducted according to the ethical standards laid down in the 1964 Declaration of Helsinki and was approved by the local ethics committee. Before participation, informed consent was obtained from all participants.

### 2.2. Clinical Assessment

Fahn's tremor rating scale was used for the clinical assessment of rest, postural, and intention tremor [16], the latter rated during the finger-nose test (0–4). In addition, spirography [16] and the nine-hole peg test (Smith & Nephew, Hull, UK) [17] were performed to estimate tremor-related disability. Overall strength of the arm was estimated by measuring handgrip using a hand-held dynamometer (JAMAR, JA Preston CO, Jackson, Mich, USA) [18]. Height, upper arm length (distance between acromion and olecranon), and forearm length (distance between processus styloideus radii and epicondylus humeralis lateralis) were measured.

### 2.3. Tendon Reflex Recording

**Figure 1:**
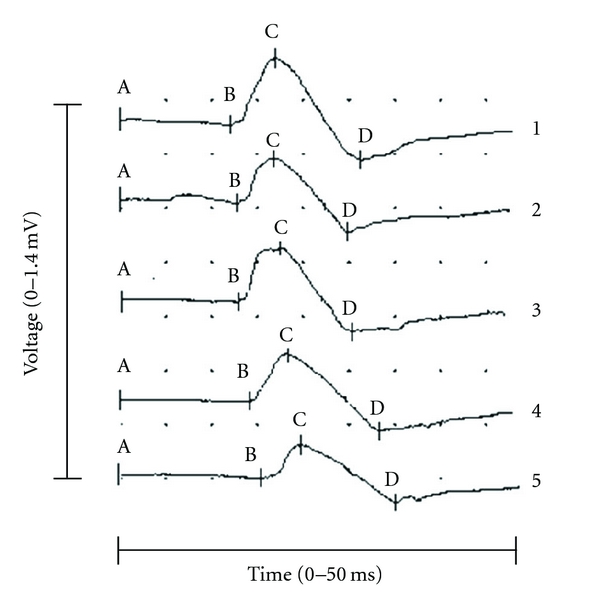
Five consecutive T-reflex responses of the biceps brachii in a healthy control subject. Latency = AB (horizontal), peak amplitude = BC (vertical), peak-to-peak amplitude = CD (vertical).

Tendon reflex responses of biceps brachii, triceps brachii, and brachioradialis muscles were bilaterally elicited using an electromechanical triggered reflex hammer connected to an electromyography (EMG) measurement device (Synergy, Oxford instruments, Surrey, UK). The reflex hammer, whose weight and handling were similar to those of typical “clinical” reflex hammers, consists of a rubber hammer with a ring contact. A firm contact between the rubber hammer and the tendon triggered a microswitch in the ring, providing a precise time baseline of the tapping. Parameters characterising the T-reflexes were onset latency, peak amplitude, and peak-to-peak amplitude and were all calculated on the basis of the surface EMG signal of the tapped muscle. The latency was measured as the time between hammer contact and the onset of the first deflection from the baseline, the peak amplitude as the amplitude between baseline and first positive peak, and the peak-to-peak amplitude as the amplitude between the positive and negative peaks. T-reflex responses of the biceps brachii of a healthy control are illustrated in Figure 1. In addition to the objective registration, an overall clinical rating (0–4) was given to the T-reflexes by the examiner. 

**Figure 2:**
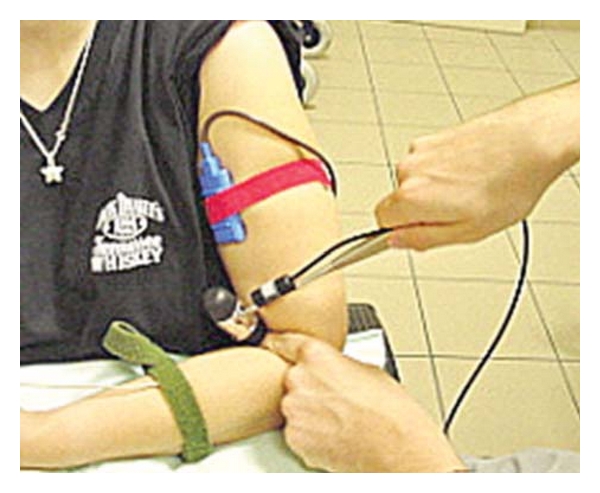
Arm and electrodes position during tapping of the biceps muscle reflex.

During the reflex assessment, subjects were comfortably seated with the forearm in midposition and supported in 90° elbow flexion (see illustration in Figure 2). All reflexes were elicited by tapping the index finger placed over the tendon. Surface EMG was registered using a bipolar electrode and a grounding electrode. The bipolar electrode was placed on the belly of the muscle, specifically at half the distance between tuberculum major and elbow fold for biceps brachii, between acromion and olecranon for triceps brachii, and 3 cm distant from the elbow fold for brachioradialis. Before tapping, muscle relaxation was controlled on the basis of the EMG signal. Reflexes were tapped 5 times on the index finger of the examiner, with an interval of approximately 5 seconds between consecutive taps. Additional taps were performed in case of accidental absent responses. Reinforcement manoeuvres were never used. Tapping force was kept as constant as possible between consecutive taps, as well as between different subjects. The physician who tapped the T-reflexes was blinded to subject group allocation to reduce subjective bias. A physiotherapist separately performed the clinical assessment. 

### 2.4. Statistical Analyses

Persons without or with insufficient number of detectable reflexes were excluded from data analyses (1 in MS-tremor, 2 in MS-no-tremor, and 2 in the healthy control group). The results were analysed in terms of the number of arms. In summary, 25 arms in 16 persons were measured in the MS-tremor group after exclusion of 3 arms because of muscle paresis and another 4 arms that did not show tremor. In the MS-no-tremor group, 24 arms of 15 persons were evaluated after exclusion of 5 arms because of muscle paresis and 1 because of difficulties in performing the clinical tasks due to a finger amputation. In the healthy control group, T-reflexes of 31 arms of 16 persons were measured after exclusion of 1 arm because of previous orthopaedic surgery. 

To investigate possible differences between the clinical characteristics of the MS-tremor and MS-no-tremor group, the unpaired *t*-test was used for examination of disease duration and the Chi-square test (*χ* ^2^) for examination of type of MS, handedness, and male-to-female ratio. The Mann-Whitney *U* tests were performed to investigate if intention tremor (finger-nose test), overall disability (EDSS), and the clinical rating of the T-reflexes differed between groups. 

Factorial ANOVAs were conducted for age, height, upper arm length, forearm length, handgrip, nine-hole peg test, and the 3 T-reflex parameters (mean latency, peak amplitude, peak-to-peak amplitude), the latter for each muscle (biceps, triceps, and brachioradialis). Bonferroni-Dunn post hoc tests were used to correct for multiple comparisons. The level of significance was set at *P* < 0.05.

## 3. Results

### 3.1. Clinical Assessment

The MS-tremor and MS-no-tremor groups did not differ significantly regarding disease duration, (*t* = −0.06; *P* = 0.96), type of MS (*χ* ^2^ = 0.16; *P* = 0.92), handedness (*χ* ^2^ = 2.13; *P* = 0.14), male-to-female ratio (*χ* ^2^ = 1.22; *P* = 0.26), and EDSS (*Z* = 1.61; *P* = 0.1). The healthy control group was on average younger than both MS groups (*F*(2,35) = 6.58; *P* < 0.01) while no differences between the MS-tremor group and the MS-no-tremor group were found. 

As intention tremor was the discriminating symptom between the 3 groups, it is not surprising that the MS-tremor group was rated significantly higher on the finger-nose test compared with the MS-no-tremor group and the healthy control group (*Z* = −5.95; *P* < 0.0001 and *Z* = −6.39; *P* < 0.0001, resp.). The finger-nose test score was not significantly different between the MS-no-tremor group and the control group (*Z* = −0.26; *P* = 0.79). In line with the finger-nose test findings, the nine-hole peg test time score was greater in the MS-tremor (86s ± 49; *F*(2,77) = 43.2; *P* < 0.0001) than in both the MS-no-tremor (32s ± 11) and the healthy control group (17.9s ± 2). Handgrip and all length outcome variables (height, upper arm, forearm) were not different among the three groups.

### 3.2. Tendon Reflex Recording

Table 2 provides an overview of the reflex parameters of the biceps brachii, triceps brachii, and brachioradialis for all 3 groups. The mean latency of the brachioradialis reflex was significantly greater in the MS-tremor compared to both the MS-no-tremor and healthy control groups (*F*(2,75) = 5.8; *P* < 0.01). Similarly, the mean latency of the biceps reflex was the greatest in the MS-tremor group and greater in the MS-no-tremor group compared to the healthy control group (*F*(2,75) = 17.2; *P* < 0.0001). The mean latency of the triceps reflex was significantly greater in the MS tremor group compared to the healthy control group (*F*(2,72) = 3.27; *P* < 0.05), but no significant differences were found between the MS-no-tremor group and either MS-tremor or control groups. 

Neither the mean peak amplitude nor the mean peak-to-peak amplitude of the biceps brachii, triceps brachii, and brachioradialis reflexes differed significantly between groups. In line with the objective findings on reflex amplitude, the clinical ratings given to the T-reflexes did not significantly differ from each other among the groups (biceps: *Z* = 0.3, *P* = 0.75; triceps: *Z* = 0.02, *P* = 0.98; brachioradialis: *Z* = −0.2, *P* = 0.82).

Additional analyses were performed to investigate if the reflex parameters changed after repeated elicitation. Repeated measures ANOVA revealed an increased latency for the brachioradialis (*F*(4,296) = 4.7, *P* < 0.001) and triceps reflex (*F*(4,264) = 2.6, *P* < 0.05) in the fifth (last) T-reflex compared to the first and/or second elicited T-reflex. However, no interaction effects of group by reflex number were found indicating that the changes were the same in all groups. For the biceps reflex, no significant difference in latency between the successive elicitations was found. In contrast to the latency findings, peak and peak-to-peak amplitudes of all three T-reflexes remained unchanged during 5 successive elicitations.

## 4. Discussion

The present study investigated upper limb tendon reflexes in MS patients with tremor in comparison with MS patients without tremor and healthy control subjects. Brachioradialis and biceps and triceps tendon reflexes were delayed in the MS patients with tremor compared to healthy control subjects. The amplitude of the upper limb tendon reflexes was not different among the groups. 

This study was generated following previous work suggesting that MS intention tremor was modulated by sensory information. Overall, intention tremor amplitude decreased after sustained peripheral cooling of the forearm [11], while it increased when muscle spindles were artificially stimulated by means of tendon vibration of the wrist extensors [12]. Given that H-reflexes are more complex to elicit in the upper limb [14], it was chosen to measure tendon reflexes, which can be easily evaluated in the clinical setting. The relevance of the tendon reflex in the present study is its sensitivity for supraspinal inhibiting and facilitating influences. It was hypothesised that T-reflexes may be delayed and enlarged in MS patients with tremor because of decreased supraspinal control due to lesions in the cerebellar system. 

All T-reflexes were evaluated while subjects were comfortably seated with the arm in 90° flexion of the elbow. This position was chosen because it is regarded as the normalised position for the elbow joint during reflex evaluation [19]. In support of this view, the amplitude of the biceps T-reflex was found to be maximal in 90° flexion compared to other elbow positions [20]. As temperature is known to have an effect on T-reflexes [21], skin temperature in each subject was carefully checked to be above 31°C. 

### 4.1. Tendon Reflex Latencies

All three upper limb reflexes were significantly delayed in the MS-tremor group compared to healthy control group. The delay could be caused by decreased supraspinal control due to lesions in the cerebellar system, or by general slowed nerve conduction velocity due to the disease of multiple sclerosis. To distinguish between both, an additional MS group without arm tremor was evaluated, however, showing similar general clinical characteristics of gender, age, disease progression, and overall disability as the MS-tremor group. The onset latency of the biceps brachii reflex was also greater in the MS-no-tremor compared to the control group; however, that of the triceps and brachioradialis muscle was not. In addition, the MS-tremor group showed significant greater reflex onset latencies for the brachioradialis and biceps muscles than the MS-no-tremor group, strongly suggesting that delayed tendon reflexes in MS patients with tremor cannot simply be attributed to decreased nerve conduction velocity due to the disease of MS. Before further interpretation of the results, other factors potentially influencing the T-reflex parameters must be discussed. First, the healthy control group was on average younger than both MS groups. It is well known that the latency of T-reflexes in the lower limb is prolonged with increasing age [22, 23]. Thus, the greater latency in both MS groups (of the same age) compared to the healthy control group (of younger age) for the biceps brachii could be due to the significant age difference. However, latency of triceps brachii and brachioradials did not differ between the MS-no-tremor group and healthy control group despite different age. In support of this, other studies examining tendon reflexes in the upper limb did not find significant correlations between age and onset latency for the biceps [20–24] and triceps brachii [24]. Another factor to be considered is the length as the latency of the biceps tendon reflex was shown to correlate with upper arm length [24] and both biceps and triceps reflex latencies correlated with height [19]. However, no differences between height, upper arm length, or forearm length were observed between the three groups in our study. One may also argue that the repetitive tapping with a short interval may have induced a postactivation depression in the T-reflex parameters [14, 25], with mean values perhaps concealing differences between the groups. Latencies of triceps brachii and brachioradialis reflexes were indeed increased during the last tendon reflex compared to the first one. However, this observation was made in all groups suggesting that it unlikely can account for any differences or similarities between the groups. The latency of biceps brachii did not change after repetitive tapping, suggesting that the mean values of latency were valid to compare between groups. Further support for the validity of the latency data is provided by comparison with other studies, showing very similar latency values for biceps and triceps reflexes recorded in healthy controls [20, 26]. 

It is noteworthy that onset latency of the triceps reflex showed less significant differences between groups compared to the brachioradialis and biceps reflexes. This could be related to a greater intersubject variability, perhaps due to a less suitable testing position or the clinical observation that this reflex is more difficult to elicit.

### 4.2. Tendon Reflex Amplitudes

In contrast to the latency data, no differences in reflex amplitude in the clinical ratings of neither biceps brachii, triceps brachii, or brachioradialis reflexes were found between subjects with or without upper limb tremor. The reflex amplitude did not decrease after repetitive tapping, similar to observations in patients with head injury when the interstimulus interval was between 1 and 10 seconds [25]. One may question the validity of the tendon reflex parameters given that the investigating physician may have changed the force of tap execution in different groups, and as such have influenced the amplitude of the T-response. First, a period of practice had preceded the actual measurements to train the physician to strike the tendon each time with similar force. Secondly, the physician was unfamiliar with the selected MS patients as he was working only very recently at the rehabilitation centre and was blinded to group allocation. Unfortunately, it cannot be excluded that tremulous movements were observed in the MS patients with tremor during the test session, for example, while taking off their watch or lifting their arm, and obviously healthy control subjects were recognized as they walked in not showing any symptoms. In this regard, the absence of major differences in reflex amplitude between all groups may actually confirm that the physician intended to strike the tendon each time with similar force. 

In contrast to latency values, many studies report a considerable intersubject and intrasubject variation in T-reflex amplitudes. Also the type of hammer used in the study and the placement of the index finger may have an effect on latency magnitude. Moreover, relatively, literature is available on tendon reflexes in upper limb muscles compared to Achilles and Patellar T-reflexes making direct comparison of absolute values difficult to perform [14].

### 4.3. Delayed Tendon Reflex and Tremor

A limitation of the study methodology is acknowledged with a manual procedure to test tendon reflexes similar to clinical practice is more variable in force application on the tendon compared to measurement in a laboratory with standardized hammer impact. Still, group differences were found, showing delayed, but not enlarged in MS patients with tremor compared to MS patients without tremor and healthy control subjects. It is hypothesised that peripheral delayed reflexes may contribute to intention tremor, however, which is an action tremor not occurring during rest conditions [1]. The tendon reflex is primarily a mono- or oligosynaptic response and is tested when the muscle is in a relaxed state, whereas stretch of an actively contracting muscle produces a more complex response with the tendon reflex often being followed by a long-latency stretch reflex. The long-latency response is reported to be increased in patients with cerebellar deficits [9, 27]. It is not thought that muscle spindle discharge frequency is changed in persons with cerebellar deficits as the range of muscle spindle sensitivity to fusimotor drive in cats was not changed when inactivating cerebellar output nuclei [28]. However, intention tremor is hypothesised to relate to malfunction of feedforward control due to cerebellar damage [5], and related excessive reliance on feedback may contribute to oscillations by means of uncontrolled alternating stretch reflexes of antagonist muscle pairs [29]. Future research should investigate peripheral reflex activity during voluntary action, for example, by means of stretch of an actively contracting muscle. 

The investigation of tendon reflexes could also be applied on other types of neurological impairments such as ataxic hemiparesis, which is an uncommon syndrome caused by lacunar cerebral infarction [30]. Reflex latencies may be different depending on whether the lacunar infarction affected the cerebellar pathways versus cerebrothalamic pathways.

## Figures and Tables

**Table 1:** Clinical characteristics of the MS-tremor group and the MS-no-tremor group.

Patient	Age (yrs)	Sex	Duration of MS (yrs)	Type of MS	EDSS	Height (cm)	Finger-nose test		Spirography	
							Left	Right	Left	Right
MS-tremor group										

1	49	F	15	SP	6	163	•	4	•	4
2	41	M	8	RR	6	174	2	2	2	2
3	53	F	35	RR	6,5	162	2	2	2	0
4	52	M	14	SP	6,5	172	2	4	4	4
5	44	F	14	SP	6,5	169	2	3	3	2
6	51	M	9	SP	7,5	170	0	2	0	3
7	51	F	15	SP	7	157	1	3	0	1
8	43	F	11	SP	8	165	2	2	1	0
9	40	F	13	SP	7	165	0	3	0	3
10	42	M	6	RR	6	163	0	2	0	3
11	46	M	8	SP	6	170	3	1	3	1
12	57	M	10	SP	6,5	174	•	1	•	0
13	33	F	6	RR	4	163	3	3	3	1
14	52	M	9	PP	6,5	190	0	2	0	4
15	48	M	24	SP	6,5	168	2	1	2	0
16	65	F	10	SP	7,5	156	2	2	2	0
17	50	M	13	PP	6	190	•	1	•	0

MS-no tremor group										

1	32	F	5	RR	4,5	184	0	0	0	0
2	55	F	12	SP	6	170	0	0	0	0
3	51	F	10	SP	6	160	0	0	0	0
4	72	M	25	RR	6	170	0	0	0	0
5	62	M	19	SP	6	180	0	0	0	0
6	58	F	23	SP	5,5	161	•	0	•	0
7	47	F	14	SP	6,5	168	0	0	0	0
8	39	M	7	RR	3,5	172	0	0	0	0
9	41	F	16	RR	6,5	160	0	0	0	0
10	45	M	12	RR	6,5	175	0	•	0	•
11	47	F	7	RR	5,5	164	0	•	0	•
12	48	M	21	SP	6,5	168	0	0	0	0
13	59	F	1	PP	6	153	0	•	0	•
14	53	F	17	RR	6,5	173	•	0	•	0
15	38	M	9	SP	7	173	0	•	0	•
16	40	F	11	SP	7	167	0	0	0	0
17	48	F	6	SP	6	164	0	0	0	0

EDSS: expanded disability status scale (0–10); RR: relapsing remitting; PP: primary progressive; SP: secondary progressive

•: no ratings due to muscle paresis or finger amputation.

**Table 2:** Mean (and standard deviation) latency, peak amplitude, and peak-to-peak amplitude of biceps, triceps, and brachioradialis reflexes for all groups.

	Biceps	Triceps	Brachioradialis
	Mean ± SD	Mean ± SD	Mean ± SD
Latency (ms)			
Controls	14.22 ± 1.43	11.38 ± 2.24	17.05 ± 1.26
MS-no-tremor	15.67 ± 1.57	12.65 ± 2.18	17.39 ± 1.79
MS-tremor	16.98 ± 2.18	13.1 ± 2.22	18.69 ± 2.38
Peak amplitude (mV)			
Controls	0.47 ± 0.35	0.24 ± 0.15	0.46 ± 0.36
MS-no-tremor	0.73 ± 0.57	0.24 ± 0.16	0.66 ± 0.64
MS-tremor	0.43 ± 0.43	0.24 ± 0.19	0.50 ± 0.45
Peak-to-peak amplitude (mV)			
Controls	0.87 ± 0.6	0.48 ± 0.27	0.78 ± 0.62
MS-no-tremor	1.19 ± 0.84	0.49 ± 0.28	1.01 ± 0.98
MS-tremor	0.76 ± 0.74	0.48 ± 0.33	0.85 ± 0.81

## References

[1] Alusi SH, Worthington J, Glickman S, Bain P (2001). A study of tremor in multiple sclerosis. *Brain*.

[2] Feys P, Maes F, Nuttin B (2005). Relationship between multiple sclerosis intention tremor severity and lesion load in the brainstem. *NeuroReport*.

[3] Hallett M (1998). Overview of human tremor physiology. *Movement Disorders*.

[4] Wolpert DM, Miall RC, Kawato M (1998). Internal models in the cerebellum. *Trends in Cognitive Sciences*.

[5] Deuschl G, Raethjen J, Lindemann M, Krack P (2001). The pathophysiology of tremor. *Muscle and Nerve*.

[6] Flament D, Vilis T, Hore J (1984). Dependence of cerebellar tremor on proprioceptive but not visual feedback. *Experimental Neurology*.

[7] Vilis T, Hore J (1977). Effects of changes in mechanical state of limb on cerebellar intention tremor. *Journal of Neurophysiology*.

[8] Richard I, Guglielmi M, Boisliveau R (1997). Load compensation tasks evoke tremor in cerebellar patients: the possible role of long latency stretch reflexes. *Neuroscience Letters*.

[9] Mauritz KH, Schmitt C, Dichgans J (1981). Delayed and enhanced long latency reflexes as the possible cause of postural tremor in late cerebellar atrophy. *Brain*.

[10] Dash BMS (1995). Role of peripheral inputs in cerebellar tremor. *Movement Disorders*.

[11] Feys P, Helsen W, Liu X (2005). Effects of peripheral cooling on intention tremor in multiple sclerosis. *Journal of Neurology, Neurosurgery and Psychiatry*.

[12] Feys P, Helsen WF, Verschueren S (2006). Online movement control in multiple sclerosis patients with tremor: effects of tendon vibration. *Movement Disorders*.

[13] Hagbarth KE, Eklund G (1968). The effects of muscle vibration in spasticity, rigidity, and cerebellar disorders. *Journal of Neurology Neurosurgery and Psychiatry*.

[14] Voerman GE, Gregorič M, Hermens HJ (2005). Neurophysiological methods for the assessment of spasticity: the Hoffman reflex, the tendon reflex, and the stretch reflex. *Disability and Rehabilitation*.

[15] Kurtzke JF (1983). Rating neurologic impairment in multiple sclerosis: an expanded disability status scale (EDSS). *Neurology*.

[16] Hooper J, Taylor R, Pentland B, Whittle IR (1998). Rater reliability of Fahn’s tremor rating scale in patients with multiple sclerosis. *Archives of Physical Medicine and Rehabilitation*.

[17] Grice KO, Vogel KA, Le V, Mitchell A, Muniz S, Vollmer MA (2003). Adult norms for a commercially available nine hole peg test for finger dexterity. *American Journal of Occupational Therapy*.

[18] Mathiowetz V, Kashman N, Volland G, Weber K, Dowe M, Rogers S (1985). Grip and pinch strength: normative data for adults. *Archives of Physical Medicine and Rehabilitation*.

[19] Péréon Y, Tich SNT, Fournier E, Genet R, Guihéneuc P (2004). Electrophysiological recording of deep tendon reflexes: normative data in children and in adults. *Neurophysiologie Clinique*.

[20] Keles I, Balci N, Beyazova M (2004). The effect of elbow position on biceps tendon reflex. *Neurology India*.

[21] Bell KR, Lehmann JF (1987). Effect of cooling on H- and T-reflexes in normal subjects. *Archives of Physical Medicine and Rehabilitation*.

[22] Kuruoglu R, Oh SJ (1993). Quantitation of tendon reflexes in normal volunteers. *Electromyography and Clinical Neurophysiology*.

[23] Koceja DM (1993). Influence of quadriceps conditioning on soleus motoneuron excitability in young and old adults. *Medicine and Science in Sports and Exercise*.

[24] Schott K, Koenig E (1991). T-wave response in cervical root lesions. *Acta Neurologica Scandinavica*.

[25] Cozens JA, Miller S, Chambers IR, Mendelow AD (2000). Monitoring of head injury by myotatic reflex evaluation. *Journal of Neurology Neurosurgery and Psychiatry*.

[26] Tarkka IM (1986). Mechanically induced reflex responses in human triceps brachii. *European Journal of Applied Physiology and Occupational Physiology*.

[27] Diener HC, Dichgans J, Bacher M, Guschlbauer B (1984). Characteristic alterations of long-loop ’reflexes’ in patients with Friedreich’s disease and late atrophy of the cerebellar anterior lobe. *Journal of Neurology Neurosurgery and Psychiatry*.

[28] Gorassini M, Prochazka A, Taylor JL (1993). Cerebellar ataxia and muscle spindle sensitivity. *Journal of Neurophysiology*.

[29] Hore J, Flament D (1986). Evidence that a disordered servo-like mechanism contributes to tremor in movements during cerebellar dysfunction. *Journal of Neurophysiology*.

[30] Arboix A, Martí-Vilaita JL (2009). Lacunar stroke. *Expert Review of Neurotherapeutics*.

